# Supplementary material for: Frequency and mortality rate following antimicrobial-resistant bloodstream infections in tertiary-care hospitals compared with secondary-care hospitals
Source: PLoS One. 2024 May 20;19(5):e0303132. doi: 10.1371/journal.pone.0303132 (PMC11104583; doi:10.1371/journal.pone.0303132)
Supplement: Attachment — Submitted filename: Referee_answer.pdf [file pone.0303132.s002.pdf]

Frequency and mortality rate following antimicrobial-resistant bloodstream infections in tertiary-care hospitals compared with secondary-care hospitals

The study aims to demonstrate an offline tool's utility in generating AMR(Antimicrobial-resistant) reports and data for secondary data analysis. Data was collected from 2012 to 2015 from 15 Tertiary-care hospitals and 34 Secondary care hospitals using AMASS. Then, a comparison is performed between both data sets, focusing on the antimicrobial-resistance infection classified by the origin, which could be from their communities or hospital origin. They found that occurrences of antimicrobial resistance bloodstream infections were twice in Tertiary-care hospitals than in Secondary care hospitals. Then, the mortality rate at Tertiary-care hospitals is higher.

My comments are:

I am impressed by the large amount of data collected during the study period; however, I recommend rejection due to the limited analysis performed and the similarities with previous studies [2,3], where the application of this R package is straightforward. AMASS has limitations, where it clearly states that data verifications should be required in the future and that the tests were conducted with only a few data sets [1].

I want to be clear; I am not stating that the approach is wrong or doubting the potential of this idea. I think it is great to have the opportunity to analyse clinical databases. I reject it because of the limitations of applying a unique package straightforwardly on a large dataset and a statistical analysis statement limited to general methods. I cannot overlook this, especially in this journal.

Additionally, some of the links do not work.

[1] Lim, C., Miliya, T., Chansamouth, V., Aung, M. T., Karkey, A., Teparrukkul, P., ... & Limmathurotsakul, D. (2020). Automating the generation of antimicrobial resistance surveillance reports: proof-of-concept study involving seven hospitals in seven countries. *Journal of medical Internet research*, *22*(10), e19762.

[2] Srisuphan, V., Klaytong, P., Rangsiwutisak, C., Tuamsuwan, K., Boonyarit, P., & Limmathurotsakul, D. (2023). Local and timely antimicrobial resistance data for local and national actions: the early implementation of an automated tool for data analysis at local hospital level in Thailand. *JAC-Antimicrobial Resistance*, *5*(4), dlad088.

[3] Goel, V., Mathew, S., Gudi, N., Jacob, A., & John, O. (2023). A scoping review on laboratory surveillance in the WHO Southeast Asia Region: Past, present and the future. *Journal of Global Health*, *13*.
