# Supplementary material for: Frequency and mortality rate following antimicrobial-resistant bloodstream infections in tertiary-care hospitals compared with secondary-care hospitals
Source: PLoS One. 2024 May 20;19(5):e0303132. doi: 10.1371/journal.pone.0303132 (PMC11104583; doi:10.1371/journal.pone.0303132)
Supplement: Attachment — Submitted filename: review PONE-D-23-31373.docx [file pone.0303132.s003.docx]

**Review of the manuscript frequency and mortality rate following antimicrobial-resistant blood stream infection in tertiary care hospital compared with the secondary care hospital.**

This study addresses an important topic comparing the burden of antimicrobial resistant bloodstream infections between tertiary care and secondary care hospitals in Thailand. The study demonstrates the utility of using automated tools like AMASS for analyzing routine surveillance data to generate useful statistics on AMR infections. However, there are some limitations that should be acknowledged:

**Major Comments**:

1. More details needed on the data analysis plan - which specific metrics were compared between tertiary and secondary care hospitals and what statistical tests were used.
2. The results section overall is well-written, but could benefit from some sub-headings to improve readability by breaking it down into logical sections.
3. One limitation as acknowledged is that the findings may not be generalizable to other LMIC settings, so it may be useful to explicitly state this in the conclusion as well.
4. The paper does not provide information on the specific criteria used to classify community-origin and hospital-origin bloodstream infections (BSI), which may affect the accuracy of the results. Based only on admission date fails to account for healthcare exposures prior to admission. This could inflate community proportions at teaching hospitals receiving many transfers e.g. Patient with MRSA blood stream infection from previous hospitalization. More granular exposure data is needed
5. Designation of the Health care originated or associated blood stream infection relies on the fact that cultured organism should not be isolated from any site other than blood in order to be classified as BSI(blood stream infection) ([www.cdc.gov/nhsn/pdfs/pscmanual/4psc_clabscurrent.pdf](http://www.cdc.gov/nhsn/pdfs/pscmanual/4psc_clabscurrent.pdf)) during the infection window period. Absence of information about the culture results during the infection window period (with same organism) from sites other than blood can overestimate the proportion of blood stream infections
6. Major potential confounders like antibiotic usage, infection control practices are not adjusted for. This limits the ability to draw causal inferences about differences between hospital types.
7. Crude mortality rates do not adjust for important risk factors and likely oversimplify differences between complex tertiary vs general hospitals. More rigorous statistical approaches are needed to control for confounding
8. Relying on routinely collected data presents various quality issues like missing/inconsistent variables, coding errors, and variability in diagnostic/prescribing practices between sites. This reduces ability to accurately measure and compare burdens.
9. No validation is reported of automated classification (e.g. AMR vs susceptible). Misclassification bias could differentially affect estimates between settings

**Minor Comments:**

1. Standardize the formatting of abbreviations - write out full forms initially before introducing abbreviations.
2. Carefully proofread the manuscript to correct any minor grammar, spelling or punctuation errors.
3. Sample sizes: with only 15 TCHs and 34 SCHs, the study may be underpowered to detect some differences, especially for less common organisms. Larger multi-year datasets could provide more robust comparisons.
4. The study does not provide information on the specific interventions or strategies that could be implemented to address the differences in AMR burden between TCHs and SCHs.
5. The term AMR is not defined clearly whether it refers to an organism resistant to multiple families of antibiotics or just one
6. Mortality measures: all-cause in-hospital deaths only capture a fraction of outcomes and underestimate AMR impacts. Longer term and attributable mortality should also be examined.
7. Proportional measures like AMR proportions do not account for differences in total case volumes between hospitals. Higher proportions may still represent lower absolute burdens at smaller hospitals. Frequencies provide more clinically meaningful comparisons but are still prone to biases from differences in populations served.
8. Focus is on bacteremia but AMR impacts many infection types. Generalizing findings may overlook reservoir/transmission dynamics across all healthcare-associated infections.

**Specific Comments:**

1. Line 95-100 : The concept provided is misinterpreted , actually the reason for expressing the rates in 100s, 1000s or even 100000 is to provide the utility of comparison of rates across different locations and cohorts regardless of the size or services of the hospital <https://www.cdc.gov/STD/Sassi/Module2/expression_of_rates.html>
2. Line 181-192: it is not clear whether the BSI with common commensals are included in the analysis or not. according to CDC, 2 or more blood samples collected with common commensals and clinical picture constitutes BSI [www.cdc.gov/nhsn/pdfs/pscmanual/4psc_clabscurrent.pdf](http://www.cdc.gov/nhsn/pdfs/pscmanual/4psc_clabscurrent.pdf)
3. Line 194-200: the statistical analysis portion need elaboration as which of the strata of have less than 5 observations. There is mention of many statistical analysis. Author needs to clarify the data type used and rationale of using the statistical analyses.
4. Line 305-319: the text is out of the context of study as the study is about the frequency , proportion and burden of blood stream infections
5. Line 324-326: there is no assessment of AMS program or the infection control program for the participating facilities. So author cannot suggest if the practices are not tailored according to the scope of services already.
6. Line 338-343: this highlight the need of robust and comprehensive data collections that captures all aspects of patient detail including the previous admission history and the previous culture results
7. Line 344-349: no reference or bases provided for the assumptions
8. Line 354-256: contradicting statement to the earlier statement in line 324-326
9. Line 390-392: it could be overestimated as well. Since only the first isolate is taken into account there is no knowledge of how well the patient responding to the antibiotics and could die of cause other than BSI.

In summary, while a pragmatic first step, the methods have limitations for making robust policy inferences about AMR burdens in different hospital contexts. Greater standardization and more sophisticated statistical analyses are needed. Prospective collection of clinical and microbiological data would allow for more rigorous analyses of differences in AMR burdens and outcomes between hospital settings. Overall the manuscript makes a useful contribution on an important topic. Addressing the comments above would further improve it. I hope these suggestions are helpful for the authors. Please feel free to let me know if you need any clarification or have additional questions!
