# Supplementary material for: Frequency and mortality rate following antimicrobial-resistant bloodstream infections in tertiary-care hospitals compared with secondary-care hospitals
Source: PLoS One. 2024 May 20;19(5):e0303132. doi: 10.1371/journal.pone.0303132 (PMC11104583; doi:10.1371/journal.pone.0303132)
Supplement: Attachment — Submitted filename: response_to_reviewers_DL.docx [file pone.0303132.s004.docx]

Journal requirements:

When submitting your revision, we need you to address these additional requirements.

1. Please ensure that your manuscript meets PLOS ONE's style requirements, including those for file naming. The PLOS ONE style templates can be found at

[https://journals.plos.org/plosone/s/file?id=wjVg/PLOSOne_formatting_sample_main_body.pdf](https://protect-eu.mimecast.com/s/Pm-XCQNJVfQ0KmCkNY9O?domain=journals.plos.org) and

[https://journals.plos.org/plosone/s/file?id=ba62/PLOSOne_formatting_sample_title_authors_affiliations.pdf](https://protect-eu.mimecast.com/s/jUkUCRMYWC62p8IqEj-G?domain=journals.plos.org)

Response: Revised as suggested.

2. We suggest you thoroughly copyedit your manuscript for language usage, spelling, and grammar. If you do not know anyone who can help you do this, you may wish to consider employing a professional scientific editing service.

Whilst you may use any professional scientific editing service of your choice, PLOS has partnered with both American Journal Experts (AJE) and Editage to provide discounted services to PLOS authors. Both organizations have experience helping authors meet PLOS guidelines and can provide language editing, translation, manuscript formatting, and figure formatting to ensure your manuscript meets our submission guidelines. To take advantage of our partnership with AJE, visit the AJE website ([http://learn.aje.com/plos/](https://protect-eu.mimecast.com/s/d0hUCV5p1cJGE1ik11ZP?domain=learn.aje.com)) for a 15% discount off AJE services. To take advantage of our partnership with Editage, visit the Editage website ([www.editage.com](https://protect-eu.mimecast.com/s/R0O3CWwO2CEJQvf1vsdI?domain=editage.com)) and enter referral code PLOSEDIT for a 15% discount off Editage services.  If the PLOS editorial team finds any language issues in text that either AJE or Editage has edited, the service provider will re-edit the text for free.

Upon resubmission, please provide the following:

The name of the colleague or the details of the professional service that edited your manuscript

A copy of your manuscript showing your changes by either highlighting them or using track changes (uploaded as a *supporting information* file)

A clean copy of the edited manuscript (uploaded as the new *manuscript* file).

Response: Revised as suggested.

3. During our evaluation of the documents provided, we noted that the data were accessed for research purposes in November 2022, but this was after the project period had expired on the IRB documents supplied. Please could you provide the ethics approval extension documents for the study. If the document is in another language, please also provide an English translation. Please note that if these documents are not included when your manuscript is resubmitted, it may be rejected.

Response: The study (MUTM 2014-017-01) was approved on 4 March 2015, and was extended for 5 rounds until 3 March 2020. The documents confirming the extension of the study approval until 2020 in English have been attached. The study was closed as per protocol of the Ethics Committee of the Faculty of Tropical Medicine, Mahidol University, that the study could be closed when all data collection had been completed. Data analysis and manuscript submission could be performed after the study closure. A letter of confirmation of the discussion about data analysis regarding this study (MUTM 2014-017-01) from Ethics Committee of the Faculty of Tropical Medicine, Mahidol University has been attached.

4. Thank you for stating the following financial disclosure:

“The study was supported by the DDC, MoPH, Thailand, and Defense Threat Reduction Agency (DTRA), U.S.. This research was funded in part by the Wellcome Trust (224681/Z/21/Z and Wellcome Trust Institutional Translational Partnership Award-MORU). CL is supported by the Wellcome Trust (106680/B/14/Z). BS is supported by a grant from the UK Department of Health and Social Care using UK aid funding managed by the Fleming Fund (R52354 CN001). For the purpose of Open Access, the author has applied a CC BY public copyright licence to any Author Accepted Manuscript version arising from this submission.”

Please state what role the funders took in the study.  If the funders had no role, please state: "The funders had no role in study design, data collection and analysis, decision to publish, or preparation of the manuscript."

If this statement is not correct you must amend it as needed.

Please include this amended Role of Funder statement in your cover letter; we will change the online submission form on your behalf.

Response: We have added a statement in both the manuscript and the cover letter to acknowledge the role of the funders in the study.

5. We noted in your submission details that a portion of your manuscript may have been presented or published elsewhere. [No. This work has not been previously published elsewhere. A pre-print version of this manuscript has been posted on medRxiv ([https://www.medrxiv.org/content/10.1101/2023.02.07.23285611v2](https://protect-eu.mimecast.com/s/aBD3CX7x3hQwg3CMQNhi?domain=medrxiv.org)).] Please clarify whether this publication was peer-reviewed and formally published. If this work was previously peer-reviewed and published, in the cover letter please provide the reason that this work does not constitute dual publication and should be included in the current manuscript.

Response: We confirm that the pre-print version posted on medRxiv was not peer-reviewed or formally published hence this work does not constitute dual publication. The medRxiv (<https://www.medrxiv.org/content/about-medrxiv>) is a free online archive for unpublished manuscripts (preprints) in medical, clinical and related health science. The current submitted manuscript has not been formally published in any platform. The below statement to clarify this is added in the cover letter:

“A pre-print version that was not peer-reviewed or formally published of this manuscript has been posted on medRxiv (https://www.medrxiv.org/content/10.1101/2023.02.07.23285611v2)”

6. We note that you have stated that you will provide repository information for your data at acceptance. Should your manuscript be accepted for publication, we will hold it until you provide the relevant accession numbers or DOIs necessary to access your data. If you wish to make changes to your Data Availability statement, please describe these changes in your cover letter and we will update your Data Availability statement to reflect the information you provide.

Response: DOIs to access our analysis data is now added in the main manuscript as below:

“The anonymous AMR surveillance reports generated from each hospital are open-access and available at <https://figshare.com/s/c028f157c18a3cc06a82>.”

7. Please include captions for your Supporting Information files at the end of your manuscript, and update any in-text citations to match accordingly. Please see our Supporting Information guidelines for more information: [http://journals.plos.org/plosone/s/supporting-information](https://protect-eu.mimecast.com/s/0YnfCYQp4F7rvPf24ZI_?domain=journals.plos.org).

Response: The captions for the supporting information files have been added at the end of the manuscript.

Additional Editor Comments:

Dear authors
Your manuscript [PONE-D-23-31373] has passed the review stage and is ready for ‎revision. ‎

Editorial comments
To ensure the Editor and Reviewers can recommend that your revised manuscript be ‎accepted, ‎‎please pay careful attention to each comment posted underneath ‎this email. This way we ‎can ‎avoid future clarifications and revisions, moving swiftly to ‎a decision.‎

‎1. Please provide a point-by-point response to the Editor and reviewer's comments
‎2. Please highlight all the amends on your manuscript with yellow color
‎3. Some grammatical and spacing errors need to be revised throughout the ‎manuscript


Editor comments:
There are significant objections to the statistical tests applied in this study.‎
As the second reviewer has pointed out, multivariable modeling ‎needs to be considered, and as the fourth reviewer has ‎pointed out, the significance of the obtained findings may be weakened ‎substantially without controlling potential confounders for investigating differences between hospital and also investigating factors associated with mortality. Please noticed that without considering advanced statistical revision I am unable to consider the manuscript further for peer review.‎
Truly yours

Response: We appreciate invaluable suggestion and feedback from the editor and reviewers. We have now added multivariable mixed-effect regression models, as suggested. After adjusted for potential confounders, our findings still show differences in the frequency and mortality rates of AMR BSI between tertiary-care and secondary-care hospitals. This additional evidence supports our conclusion that targets of and resources for antimicrobial stewardship and infection control programs in LMICs might need to be tailored based on hospital setting, as burden of AMR infections might differ by hospital setting. We have added the results from the multivariable models in both the main text of the manuscript and supporting documents.

[Note: HTML markup is below. Please do not edit.]

Reviewers' comments:

Reviewer's Responses to Questions

**Comments to the Author**

1. Is the manuscript technically sound, and do the data support the conclusions?

The manuscript must describe a technically sound piece of scientific research with data that supports the conclusions. Experiments must have been conducted rigorously, with appropriate controls, replication, and sample sizes. The conclusions must be drawn appropriately based on the data presented.

Reviewer #1: Yes

Reviewer #2: Partly

Reviewer #3: Partly

Reviewer #4: Partly

2. Has the statistical analysis been performed appropriately and rigorously?

Reviewer #1: Yes

Reviewer #2: No

Reviewer #3: Yes

Reviewer #4: No

3. Have the authors made all data underlying the findings in their manuscript fully available?

The [PLOS Data policy](https://protect-eu.mimecast.com/s/ScFmCZwO5C6JwyIJtFv5?domain=plosone.org) requires authors to make all data underlying the findings described in their manuscript fully available without restriction, with rare exception (please refer to the Data Availability Statement in the manuscript PDF file). The data should be provided as part of the manuscript or its supporting information, or deposited to a public repository. For example, in addition to summary statistics, the data points behind means, medians and variance measures should be available. If there are restrictions on publicly sharing data—e.g. participant privacy or use of data from a third party—those must be specified.

Reviewer #1: Yes

Reviewer #2: Yes

Reviewer #3: Yes

Reviewer #4: Yes

4. Is the manuscript presented in an intelligible fashion and written in standard English?

PLOS ONE does not copyedit accepted manuscripts, so the language in submitted articles must be clear, correct, and unambiguous. Any typographical or grammatical errors should be corrected at revision, so please note any specific errors here.

Reviewer #1: Yes

Reviewer #2: Yes

Reviewer #3: Yes

Reviewer #4: Yes

5. Review Comments to the Author

Please use the space provided to explain your answers to the questions above. You may also include additional comments for the author, including concerns about dual publication, research ethics, or publication ethics. (Please upload your review as an attachment if it exceeds 20,000 characters)

Reviewer #1: Thanks to the research team for their valuable work. Please find my comments below:
1. In lines 220 to 227, you mentioned the hospitals included in the study, stating that out of 96 hospitals, 46 were excluded under certain conditions, and 49 studies were analyzed in your research, while it should have been 50. Please provide an explanation for this discrepancy regarding one study.

Response: We approached 96 hospitals and 95 of them agreed to participate in the study (Line 217). Of these 95 hospitals, 46 were excluded and 49 were analysed. We revised the sentence as below to clarify this:

“Ninety-five (99%) hospitals, out of 96 Thai hospitals that we approached, agreed to participate in the study.”

2. In the statistical section, it is necessary to clarify which variables have fixed effect(s) and which have random effect(s) in your mixed-effects logistic model.

Response: The lines below are now revised and added in manuscript to clarify mixed-effect models.

“We estimated the magnitude of differences in proportions and mortality of AMR BSI between SCHs and TCHs using mixed-effect logistic regression models for patients nested within hospital using the STATA command xtlogit. We estimated the magnitude of differences in frequency and mortality rate of AMR BSI between SCHs and TCHs using mixed-effect Poisson regression models for patients nested within hospital using the STATA command xtpoisson. We used mixed-effect models to estimate the fixed effects of hospital type while taking account for the random effects of patient within the same hospital. Multivariable mixed-effect models were also performed to control for other variables, including blood culture utilization rates of the hospitals and the health region where the hospitals located. These variables were included in the multivariable models because they could be associated with the hospital type and the outcome variables.”

3. Please be aware that p-values only indicate the level of significance, not the strength of the relationship. Therefore, consider this in all your tables and replace all values of <0.0001 with <0.001. NOTICE: "When p-values are displayed as very small numbers (e.g., 0.0001), it can be challenging for readers to quickly grasp their significance. It's more common to use the conventional format of displaying p-values as three decimal places (e.g., 0.001) for ease of interpretation."

Response: P-values in the text have been revised as suggested.

4. The quality of your figures is significantly low, or they did not meet the desired quality in the submitted version, and I could not extract any useful information from them.. Please ensure that the figures meet the required quality standards or provide better versions in the submitted manuscript.

Response: Figures with improved resolutions have been uploaded. The figures with improved resolutions can be downloaded from the PLoS website using the links on the PDF.

5. In the references section, you've used reports as sources. Please include relevant links for each reference to facilitate readers' access to the sources in your article.

Response: The links for references to reports have been added.

Reviewer #2: I am impressed by the large amount of data collected during the study period 2012 to 2015; however, I recommend rejection due to the limited analysis performed and the similarities with previous studies.

Response: Additional multivariable models are now added to control for variables that define the characteristics of the hospitals. The findings from the multivariable models have been added in both the main text of the manuscript and supporting documents. The results from the multivariable models still suggested differences in the observed frequency and mortality rates of AMR BSI between tertiary-care and secondary-care hospitals. The additional evidence supports our conclusion that the targets of and resources for antimicrobial stewardship and infection control programs in LMICs might need to be tailored based on hospital type and size, as burden of AMR infections might differ by hospital setting.

In addition, there are few studies comparing frequency and mortality rate following antimicrobial-resistant infections between tertiary-care hospitals and secondary-care hospitals in LMICs.

Reviewer #3: Dear Authors

Kindly address the following few concerns

Line 154 correct the grammatical error " local importance" instead of "locally importance."

Response: Revised as suggested.

Line 215, second sentence is unclear, kindly revise.

Response: Line 215 has been revised as below:

“Consent was not sought from the individual patients as this was a retrospective study. This approach was approved by the Ethical and Scientific Review committees.”

LINE 398 "This is likely occurring in other LMICs” as part of the conclusion is too much of extrapolation from the current study under review. Rephrasing it to eg “This may be situation in other LMICs, or This might also be occurring in other LMICs” will be more scientifically acceptable.

Response: Revised as suggested.

Additional concerns
1. please include a section/paragraph that describes challenges or weakness or limitations of this study.

Response: A section that discuss the limitations of this study has been revised and expanded as below:

“Our study has some limitations. Firstly, the findings from the study may not be generalisable to all LMICs. The differences in proportions, frequencies, all-cause in-hospital mortality and all-cause in-hospital mortality rates following AMR BSI in SCHs and TCHs could be confounded by many factors such as difference in patient characteristics, diagnostic stewardship (particularly blood culture utilization^35,36^) and patient management (including AMS and IPC). For example, the differences in frequency and in-hospital mortality rate observed in our study could be confounded by the differences in the case mix between SCHs and TCHs. It is also possible that the AMS and IPC practices in Thailand are better in SCHs compared to TCHs due to various reasons. These data were not available in our study. To understand the size of effect caused by the difference in hospital type alone, further studies using detailed clinical data, AMS and IPC data, and rigorous statistical approaches are needed. It is also possible that the AMS and IPC has already been tailored base on hospital size and type already. Regardless, our findings emphasize the need for further evaluation and improvement of all actions against AMR infections (including AMS and IPC) in TCHs, going beyond the current practices. Secondly, the sample size of SCHs and TCHs included in this study is small, and this may have limited the power to detect differences for organisms that are less predominate such as CRKP in the study setting. Thirdly, in this study we only included patients who were hospitalised and in-hospital mortality. Patients who had blood cultures taken either at community hospitals or the study hospitals but not hospitalised at the study hospitals were not included in the analysis. Fourthly, the mortality and mortality rate associated with AMR BSI reported are all-cause in-hospital mortality and mortality rate, and could be underestimated because some people, in the study area, preferred to die at home and were discharged against advice. The all-cause in-hospital mortality could also overestimate the impact of AMR infection. This is because a proportion of patients with AMR infection might die of other causes after the successful treatment of the AMR infection. This secondary data analysis was not designed to answer causal question but to demonstrate the potentials of using AMASS as a tool to empower routine AMR surveillance data understanding and sharing. Fifthly, the distinction between primary and secondary BSIs cannot be made without detailed, linked clinical and microbiology data. The BSIs in this study include both primary and secondary BSIs. Future surveillance systems should consider innovative methods that can integrate such information to ensure that these distinctions can be accurately made in LMICs. In addition, future studies with robust and comprehensive data collections that can capture all aspect of patient details, including the previous admission history and the previous culture results, the current diagnostic stewardship, AMS and IPC, and other confounding factors, are still needed.

2. Are the patients' demographics accessible? We could make more meaningful inference from the comparative analysis with the demographic data. If this is not available, kindly include it in the weakness section.

Response: Thank you for the suggestion. Individual-patient level data is not available for the analysis. This limitation has been revised as shown in the response above.

Reviewer #4: Review of the manuscript frequency and mortality rate following antimicrobial-resistant blood stream infection in tertiary care hospital compared with the secondary care hospital.
This study addresses an important topic comparing the burden of antimicrobial resistant bloodstream infections between tertiary care and secondary care hospitals in Thailand. The study demonstrates the utility of using automated tools like AMASS for analyzing routine surveillance data to generate useful statistics on AMR infections. However, there are some limitations that should be acknowledged:

Major Comments:
1. More details needed on the data analysis plan - which specific metrics were compared between tertiary and secondary care hospitals and what statistical tests were used.

Response: We appreciate your suggestion. The statistical analysis has been revised for clarity as below:

“We compared proportion, frequency, mortality and mortality rate of AMR BSI between SCHs and TCHs for each pathogen of interest. We compared AMR proportions and mortality using Chi-square or Fisher’s exact test when small samples (<5 observations in any one strata) and measurements from continuous variables using the Krustal-Wallis test. We estimated the magnitude of differences in proportions and mortality of AMR BSI between SCHs and TCHs using mixed-effect logistic regression models for patients nested within hospital using the STATA command xtlogit. We estimated the magnitude of differences in frequency and mortality rate of AMR BSI between SCHs and TCHs using mixed-effect Poisson regression models for patients nested within hospital using the STATA command xtpoisson. We used mixed-effect models to estimate the fixed effects of hospital type while taking account for the random effects of patient within the same hospital. Multivariable mixed-effect models were also performed to control for other variables, including blood culture utilization rates of the hospitals and the health region where the hospitals located. These variables were included in the multivariable models because they could be associated with the hospital type and the outcome variables. We then calculated total number and in-hospital mortality of patients with culture-confirmed notifiable bacterial diseases under evaluation. We used STATA (version 17.0; College Station, Texas) for the final statistical analyses and R version 4.0.5 for figures.”

Details of each metric has been described in the Definitions in the Method section.

2. The results section overall is well-written, but could benefit from some sub-headings to improve readability by breaking it down into logical sections.

Response: We appreciate your suggestion. The Results section has now been structured into several subsections; including Baseline Demographics, Proportion of AMR BSI, Frequency of AMR BSI, Mortality following AMR BSI, and Mortality Rate following AMR BSI. This organization is based on the analysis plan outlined in the Methods section and follows a logical progression. It is generally understood by readers that the proportion of AMR BSI (%) serves as the primary indicator of AMR, followed by the frequency as the next indicator. The mortality (%) and mortality rate following BSI are presented subsequently, adhering to a similar logical sequence.

3. One limitation as acknowledged is that the findings may not be generalizable to other LMIC settings, so it may be useful to explicitly state this in the conclusion as well.

Response: We appreciate your suggestion. The conclusion in the main text has been revised as below:

“In conclusion, we observed that the burden of AMR infections in TCHs is higher than that in SCHs in Thailand. This might also be occurring in other LMICs. Our results support similar evaluations in other LMICs and highlight the importance of tailoring infection control strategies based on hospital size and type, particularly when significant differences in the burden are observed.”

The conclusion in the abstract has been revised as below:

“In conclusion, the burden of AMR infections in some LMICs might differ by hospital type and size. In those countries, activities and resources for antimicrobial stewardship and infection control programs might need to be tailored based on hospital setting.”

4. The paper does not provide information on the specific criteria used to classify community-origin and hospital-origin bloodstream infections (BSI), which may affect the accuracy of the results. Based only on admission date fails to account for healthcare exposures prior to admission. This could inflate community proportions at teaching hospitals receiving many transfers e.g. Patient with MRSA blood stream infection from previous hospitalization. More granular exposure data is needed

Response: Thank you for the comment on the criteria to classify community-origin and hospital-origin bloodstream infections (BSI). We cannot agree more that the classification is not always straightforward especially without granular individual-patient level data. However, to ensure the AMR surveillance reports can be consistent and performed automatically (within minimal data collection efforts), we followed the WHO GLASS classification criteria of using the two calendar days. We have added the sentences as below to clarify the classification criteria.

“Community-origin BSI was defined for patients with first positive blood specimens in the hospital taken within the first two calendar days of admission with calendar day one equal to the day of admission.^20^ Patients with first positive blood specimens taken after the first two calendar days were categorized as cases of hospital-origin BSI.”

5. Designation of the Health care originated or associated blood stream infection relies on the fact that cultured organism should not be isolated from any site other than blood in order to be classified as BSI(blood stream infection) ([www.cdc.gov/nhsn/pdfs/pscmanual/4psc_clabscurrent.pdf](https://protect-eu.mimecast.com/s/DptwC1Y15C4V0KHWLhSu?domain=cdc.gov)) during the infection window period. Absence of information about the culture results during the infection window period (with same organism) from sites other than blood can overestimate the proportion of blood stream infections

Response: Thank you for highlighting the limitation of defining bloodstream infections without clinical data in resource-limited settings where clinical data are not available to refine the distinction between primary and secondary bloodstream infections. We have now added the sentence as below to highlight this limitation.

“Fifthly, the distinction between primary and secondary BSIs cannot be made without detailed, linked clinical and microbiology data. The BSIs in this study include both primary and secondary BSIs. Future surveillance systems should consider innovative methods that can integrate such information to ensure that these distinctions can be accurately made in LMICs.”

6. Major potential confounders like antibiotic usage, infection control practices are not adjusted for. This limits the ability to draw causal inferences about differences between hospital types.

Response: Thank you for the feedback. We agree with the reviewer that causal conclusions cannot be made using the analysed data, and indeed the study was not designed to answer causal questions. We designed the study aiming to empower re-used of routinely collected data and to showcase how AMR surveillance data can be shared and re-used to generate new hypotheses. To clarify the limitation of the study we have revised the discussion as below:

“To understand the size of effect caused by the difference in hospital type alone, further studies using detailed clinical data, AMS and IPC data, and rigorous statistical approaches are needed. It is also possible that the AMS and IPC has already been tailored base on hospital size and type already. Regardless, our findings emphasize the need for further evaluation and improvement of all actions against AMR infections (including AMS and IPC) in TCHs, going beyond the current practices.”

“This secondary data analysis was not designed to answer causal question but to demonstrate the potentials of using AMASS as a tool to empower routine AMR surveillance data sharing..”

7. Crude mortality rates do not adjust for important risk factors and likely oversimplify differences between complex tertiary vs general hospitals. More rigorous statistical approaches are needed to control for confounding

Response: Thank you for the feedback. Indeed the study was not designed to answer causal questions and not to suggest that admitting patients to tertiary hospital would result in higher mortality rate. The discussion has been revised for clarity as below,

“Firstly, the findings from the study may not be generalisable to all LMICs. The differences in proportions, frequencies, all-cause in-hospital mortality and all-cause in-hospital mortality rates following AMR BSI in SCHs and TCHs could be confounded by many factors such as difference in patient characteristics, diagnostic stewardship (particularly blood culture utilization35,36) and patient management (including AMS and IPC). For example, the differences in frequency and in-hospital mortality rate observed in our study could be confounded by the differences in the case mix between SCHs and TCHs. It is also possible that the AMS and IPC practices in Thailand are better in SCHs compared to TCHs due to various reasons. These data were not available in our study. To understand the size of effect caused by the difference in hospital type alone, further studies using detailed clinical data, AMS and IPC data, and rigorous statistical approaches are needed. It is also possible that the AMS and IPC has already been tailored base on hospital size and type already. Regardless, our findings emphasize the need for further evaluation and improvement of all actions against AMR infections (including AMS and IPC) in TCHs, going beyond the current practices.” and “In addition, future studies with robust and comprehensive data collections that can capture all aspect of patient details, including the previous admission history and the previous culture results, the current diagnostic stewardship, AMS and IPC, and other confounding factors, are still needed.”

8. Relying on routinely collected data presents various quality issues like missing/inconsistent variables, coding errors, and variability in diagnostic/prescribing practices between sites. This reduces ability to accurately measure and compare burdens.

Response: We agree with the reviewer that comparison in AMR burden between different settings are difficult and need to be made with cautions. Details of quality issue for laboratory in Thailand has been added as shown in the response above. The details of the data validation have been added as shown in the response above. A sentence to highlight the need for rigorous studies have been expanded as below:

“In addition, future studies with robust and comprehensive data collections that can capture all aspect of patient details, including the previous admission history and the previous culture results, the current diagnostic stewardship, AMS and IPC, and other confounding factors, are still needed.”

9. No validation is reported of automated classification (e.g. AMR vs susceptible). Misclassification bias could differentially affect estimates between settings

Response: The automated classification was based on the results exported from the microbiology laboratory of each hospital. Thailand has strong quality control for the microbiology laboratories in public hospitals nationwide. The following sentences and references have been noted in the Method section,

“In each province, there is at least one SCH or TCH,^17^ equipped with a microbiology laboratory capable of performing bacterial culture using standard methodologies for bacterial identification and susceptibility testing provided by the Bureau of Laboratory Quality and Standards, MoPH, Thailand.^18^”

We also validated the summary data and the reports generated by the AMASS intensively. The following sentences have been added for clarity,

“The reports generated by the AMASS were validated using two methods: (a) checking data verification log files generated by the AMASS whether all information was imported accurately (e.g. total number of specimens, total number of hospital admissions, number of missing values, total number of isolates per organism in the raw microbiology data file, total number of antibiotics being tested)^16^ and (b) comparing the summary data and reported generated by the AMASS with data generated from manual calculations obtained from complete line listing of several organisms.”

Minor Comments:
1. Standardize the formatting of abbreviations - write out full forms initially before introducing abbreviations.

Response: Revised as suggested.

2. Carefully proofread the manuscript to correct any minor grammar, spelling or punctuation errors.

Response: Performed as suggested.

3. Sample sizes: with only 15 TCHs and 34 SCHs, the study may be underpowered to detect some differences, especially for less common organisms. Larger multi-year datasets could provide more robust comparisons.

Response: Thank you for the feedback. We did use multi-year data. The limitation has been noted as below,

“Secondly, the sample size of SCHs and TCHs included in this study is small, and this may have limited the power to detect differences for organisms that are less predominate such as CRKP in the study setting.”

4. The study does not provide information on the specific interventions or strategies that could be implemented to address the differences in AMR burden between TCHs and SCHs.

Response: Thank you for the feedback. The recommendation on intervention or strategies are not within the scope of our studies. We believe that further studies on intervention, strategies and implementation are needed.

5. The term AMR is not defined clearly whether it refers to an organism resistant to multiple families of antibiotics or just one

Response: In this study, the term AMR is for the single antibiotic as per WHO priority list of AMR pathogens. The following section in the manuscript has described our definition,

“AMR BSI is defined as a case of infection in patients with blood culture positive for CRAB, CRPA, 3GCREC, 3GCRKP, CREC, CRKP or MRSA. Non-AMR BSI is defined as cases of infection in patients who had blood culture positive for carbapenem-susceptible *A. baumannii* (CSAB), carbapenem-susceptible *P. aeruginosa* (CSPA), third-generation cephalosporin-susceptible *E. coli* (3GCSEC) or *K. pneumoniae* (3GCSKP), or methicillin-susceptible *S. aureus* (MSSA).”

6. Mortality measures: all-cause in-hospital deaths only capture a fraction of outcomes and underestimate AMR impacts. Longer term and attributable mortality should also be examined.

Response: Thank you for the feedback. We agree that further studies with rigorous data on longer term and attributable mortality using all detailed clinical data are needed. The limitation has been revised extensively as shown in the response above.

7. Proportional measures like AMR proportions do not account for differences in total case volumes between hospitals. Higher proportions may still represent lower absolute burdens at smaller hospitals. Frequencies provide more clinically meaningful comparisons but are still prone to biases from differences in populations served.

Response: We thank the reviewer for the comment and agree that frequencies are more meaningful. The limitation that it is prone to biases from differences in case mix have been added as below,

“For example, the differences in frequency and in-hospital mortality rate observed in our study could be confounded by the differences in the case mix between SCHs and TCHs. It is also possible that the AMS and IPC practices in Thailand are better in SCHs compared to TCHs due to various reasons. These data were not available in our study. To understand the size of effect caused by the difference in hospital type alone, further studies using detailed clinical data, AMS and IPC data, and rigorous statistical approaches are needed. It is also possible that the AMS and IPC has already been tailored base on hospital size and type already. Regardless, our findings emphasize the need for further evaluation and improvement of all actions against AMR infections (including AMS and IPC) in TCHs, going beyond the current practices.”

8. Focus is on bacteremia but AMR impacts many infection types. Generalizing findings may overlook reservoir/transmission dynamics across all healthcare-associated infections.

Response: We thank the reviewer for the comment and agree that it is beyond the scope of the current study to inform transmission dynamic and identify reservoir across different types of healthcare-associated infections.

Specific Comments:
1. Line 95-100 : The concept provided is misinterpreted , actually the reason for expressing the rates in 100s, 1000s or even 100000 is to provide the utility of comparison of rates across different locations and cohorts regardless of the size or services of the hospital [https://www.cdc.gov/STD/Sassi/Module2/expression_of_rates.html](https://protect-eu.mimecast.com/s/jBxZC2g46hYM4Af8e0w6?domain=cdc.gov)
Response: Thank you for the comment. The following sentence has now been revised for clarity about this point:

“The AMR frequencies, typically expressing per 1000, 1,000 or 100,000, are also commonly used to monitor, evaluate and compare the AMR burden between hospitals or survey sites regardless of the size or services of the hospital.”

2. Line 181-192: it is not clear whether the BSI with common commensals are included in the analysis or not. according to CDC, 2 or more blood samples collected with common commensals and clinical picture constitutes BSI [www.cdc.gov/nhsn/pdfs/pscmanual/4psc_clabscurrent.pdf](https://protect-eu.mimecast.com/s/DptwC1Y15C4V0KHWLhSu?domain=cdc.gov)

Response: In the current manuscript, we analysed the organisms in WHO priority list including CRAB and carbapenem-resistant *Pseudomonas aeruginosa* (CRPA), third-generation cephalosporin-resistant *Escherichia coli* (3GCREC) and *Klebsiella pneumoniae* (3GCRKP), carbapenem-resistant *E. coli* (CREC), carbapenem-resistant *K*. *pneumoniae* (CRKP) and MRSA (Line 152-157). Common commensals such as Staphylococcus epidermis were not included in the analysis.

3. Line 194-200: the statistical analysis portion need elaboration as which of the strata of have less than 5 observations. There is mention of many statistical analysis. Author needs to clarify the data type used and rationale of using the statistical analyses.

Response: We thank the reviewer for the comment. The statistical analysis section has been revised as shown in the response above..

4. Line 305-319: the text is out of the context of study as the study is about the frequency , proportion and burden of blood stream infections

Response: We thank the reviewer for the comment. We revised the manuscript for clarity that this is also within the aim of the study to utilize AMASS on retrospective data as below,

“The aim of this study was to examine the burden of AMR BSI in TCHs and compare that with SCHs using AMASS^15^ on the microbiology and hospital admission data from 2012 and 2015 in Thailand. We also examine the burden of culture-confirmed notifiable bacterial diseases under evaluation using the reports generated by the AMASS.”

The Method section has also been revised accordingly.

5. Line 324-326: there is no assessment of AMS program or the infection control program for the participating facilities. So author cannot suggest if the practices are not tailored according to the scope of services already.

Response: We thank the reviewer for the comment. The following sentences have been added into the discussion for clarity,

“It is also possible that the AMS and IPC has already been tailored base on hospital size and type already. Regardless, our findings emphasize the need for further evaluation and improvement of all actions against AMR infections (including AMS and IPC) in TCHs, going beyond the current practices.”

6. Line 338-343: this highlight the need of robust and comprehensive data collections that captures all aspects of patient detail including the previous admission history and the previous culture results

Response: We thank the reviewer for the comment. The following sentences have been added into the discussion for clarity,

“In addition, future studies with robust and comprehensive data collections that can capture all aspect of patient details, including the previous admission history and the previous culture results, the current diagnostic stewardship, AMS and IPC, and other confounding factors, are still needed.”

7. Line 344-349: no reference or bases provided for the assumptions

Response: References 26 and 27 have been provided for the assumptions.

8. Line 354-256: contradicting statement to the earlier statement in line 324-326

Response: The previous line 354-356 is for all-cause in-hospital mortality (%) while the previous line 324-326 is for all-cause in-hospital mortality rate (per 100,000 admissions and per 100,000 patient-days). The sentences have been revised for clarity.

9. Line 390-392: it could be overestimated as well. Since only the first isolate is taken into account there is no knowledge of how well the patient responding to the antibiotics and could die of cause other than BSI.

Response: We thank the reviewer for the comment. The following sentences have been added into the discussion for clarity,

“The all-cause in-hospital mortality could also overestimate the impact of AMR infection. This is because a proportion of patients might die of other causes after the successful treatment of the infection.”

In summary, while a pragmatic first step, the methods have limitations for making robust policy inferences about AMR burdens in different hospital contexts. Greater standardization and more sophisticated statistical analyses are needed. Prospective collection of clinical and microbiological data would allow for more rigorous analyses of differences in AMR burdens and outcomes between hospital settings. Overall the manuscript makes a useful contribution on an important topic. Addressing the comments above would further improve it. I hope these suggestions are helpful for the authors. Please feel free to let me know if you need any clarification or have additional questions!

Response: Thank you for the feedback and helpful suggestions. We have now added additional multivariable models to adjust for variables that define characteristic of hospital type and outcomes. We cannot agree more with the reviewer that prospective collections of clinical and microbiology data would allow for rigorous analyses. We hope our study can be a showcase for how routinely collected microbiology and routine clinical data can be used to generate hypothesis and inform directions of future data collection and studies.

6. PLOS authors have the option to publish the peer review history of their article ([what does this mean?](https://protect-eu.mimecast.com/s/y8AkC3jR7SknPVHzkF8c?domain=journals.plos.org)). If published, this will include your full peer review and any attached files.


If you choose “no”, your identity will remain anonymous but your review may still be made public.


**Do you want your identity to be public for this peer review?** For information about this choice, including consent withdrawal, please see our [Privacy Policy](https://protect-eu.mimecast.com/s/E-jwC4kmgIVXnGHLIjfA?domain=plos.org).

Reviewer #1: No

Reviewer #2: No

Reviewer #3: No

Reviewer #4: No
