# Supplementary material for: Frequency and mortality rate following antimicrobial-resistant bloodstream infections in tertiary-care hospitals compared with secondary-care hospitals
Source: PLoS One. 2024 May 20;19(5):e0303132. doi: 10.1371/journal.pone.0303132 (PMC11104583; doi:10.1371/journal.pone.0303132)
Supplement: Attachment — Submitted filename: response_to_reviewers_2_DL.docx [file pone.0303132.s005.docx]

Re: PONE-D-23-31373R1

Frequency and mortality rate following antimicrobial-resistant bloodstream infections in tertiary-care hospitals compared with secondary-care hospitals

PLOS ONE

Dear Editor and Reviewers,

We would like to thank the editor and all the reviewers for their time reading and commenting on our manuscript. We have revised the manuscript based on the feedback. In brief, the conclusion in the Abstract and Discussion sections are revised as suggested by the Editor. Also, frequencies, p-values, and mortality rates are included in the Abstract section as recommended by the Editor. The statistical analysis section is revised to improve clarity.

We very much appreciate the suggestions and made changes in our manuscript as below:

Editor comments:

Abstract:‎
‎Add corresponding statistics in the “results” section for the data presented in ‎the abstract section. Address the following shortcomings: ‎
a. “The frequencies of hospital-origin AMR BSI per 100,000 patient-days at risk ‎in TCHs were about twice that in SCHs for most pathogens under evaluation.”; ‎add frequencies and p. value. ‎

Response: The frequencies and p values have been added as below:

“(for carbapenem-resistant Acinetobacter baumannii [CRAB]: 18.6 vs. 7.0, incidence rate ratio 2.77; 95%CI 1.72-4.43, p<0.001; for carbapenem-resistant Pseudomonas aeruginosa [CRPA]: 3.8 vs. 2.0, p=0.0073; third-generation cephalosporin resistant Escherichia coli [3GCREC]: 12.1 vs. 7.0, p<0.001; third-generation cephalosporin resistant Klebsiella pneumoniae [3GCRKP]: 12.2 vs. 5.4, p<0.001; carbapenem-resistant K. pneumoniae [CRKP]: 1.6 vs. 0.7, p=0.045; and methicillin-resistant Staphylococcus aureus [MRSA]: 5.1 vs. 2.5, p=0.0091).”

b. “All-cause in-hospital mortality (%) following hospital-origin AMR BSI was
not significantly different between TCHs and SCHs.”; add p. value.‎

Response: The p-values have been added.

c. “However, due to the higher frequencies, all-cause in-hospital mortality rates ‎following hospital-origin AMR BSI per 100,000 patient-days at risk were ‎considerably higher in TCHs for most pathogens.”; remove “However”; add ‎frequencies and p. value.‎

Response: “However” and “For example” are now removed. The frequencies and p values are reported as below:

“Due to the higher frequencies, all-cause in-hospital mortality rates following hospital-origin AMR BSI per 100,000 patient-days at risk were considerably higher in TCHs for most pathogens (for CRAB: 10.2 vs. 3.6, mortality rate ratio 2.77; 95%CI 1.71 to 4.48, p<0.001; CRPA: 1.6 vs. 0.8; p=0.020; 3GCREC: 4.0 vs. 2.4, p=0.009; 3GCRKP, 4.0 vs. 1.8, p<0.001; CRKP: 0.8 vs. 0.3, p=0.042; and MRSA: 2.3 vs. 1.1, p=0.023).”

d. the following statement need corrections: ‎
‎“For example, the all-cause in-hospital mortality rate ...”; remove “For example, ”‎

Response: “For example” are now removed.

e. Conclusion should be improved (both abstract and main text). It is a fact that AMR is varied in different ‎hospital settings. Based on the results of your study, you should highlight the ‎important indicators that found to be clinically more different and suggest ‎appropriate action to address issues.‎
Response: The conclusion is now improved as below in the Abstract and in the Discussion:

“The frequency and in-hospital mortality rate of hospital-origin AMR BSI are important indicators and should be routinely measured to monitor the burden of AMR in every hospital with microbiology laboratories in LMICs.”

[Note: HTML markup is below. Please do not edit.]

Reviewers' comments:

Reviewer's Responses to Questions

**Comments to the Author**

1. If the authors have adequately addressed your comments raised in a previous round of review and you feel that this manuscript is now acceptable for publication, you may indicate that here to bypass the “Comments to the Author” section, enter your conflict of interest statement in the “Confidential to Editor” section, and submit your "Accept" recommendation.

Reviewer #1: (No Response)

Reviewer #3: All comments have been addressed

Reviewer #4: All comments have been addressed

2. Is the manuscript technically sound, and do the data support the conclusions?

The manuscript must describe a technically sound piece of scientific research with data that supports the conclusions. Experiments must have been conducted rigorously, with appropriate controls, replication, and sample sizes. The conclusions must be drawn appropriately based on the data presented.

Reviewer #1: Yes

Reviewer #3: Yes

Reviewer #4: Yes

3. Has the statistical analysis been performed appropriately and rigorously?

Reviewer #1: No

Reviewer #3: Yes

Reviewer #4: Yes

4. Have the authors made all data underlying the findings in their manuscript fully available?

The [PLOS Data policy](https://url.uk.m.mimecastprotect.com/s/9MpxCMED0FZP26HW3vXM?domain=plosone.org) requires authors to make all data underlying the findings described in their manuscript fully available without restriction, with rare exception (please refer to the Data Availability Statement in the manuscript PDF file). The data should be provided as part of the manuscript or its supporting information, or deposited to a public repository. For example, in addition to summary statistics, the data points behind means, medians and variance measures should be available. If there are restrictions on publicly sharing data—e.g. participant privacy or use of data from a third party—those must be specified.

Reviewer #1: No

Reviewer #3: Yes

Reviewer #4: Yes

5. Is the manuscript presented in an intelligible fashion and written in standard English?

PLOS ONE does not copyedit accepted manuscripts, so the language in submitted articles must be clear, correct, and unambiguous. Any typographical or grammatical errors should be corrected at revision, so please note any specific errors here.

Reviewer #1: Yes

Reviewer #3: Yes

Reviewer #4: Yes

6. Review Comments to the Author

Please use the space provided to explain your answers to the questions above. You may also include additional comments for the author, including concerns about dual publication, research ethics, or publication ethics. (Please upload your review as an attachment if it exceeds 20,000 characters)

Reviewer #1: Your statistical section leaves me feeling disconnected for a couple of reasons. Firstly, there's no mention of demographic variables for the patients. Was this data unavailable or deemed unnecessary for your model? Secondly, the confounding variables you adjusted for aren't specified. Were these variables shared between patients and hospitals? Were they chosen based on their relevance to patients or hospitals? The only insights provided are tucked away in the supplementary file, in the footer of the tables.

Response: We would like to thank the reviewer for highlighting the potential confusing points in our manuscript. No individual patient-level data on demographic variables was collected. Our study is a secondary data analysis, which involved extracting aggregated statistics on AMR burden from routine AMR surveillance report generated by the 49 local hospitals. The sentence below is added to clarify:

“This secondary data analysis was not designed to answer causal question but to demonstrate the potentials of using AMASS as a tool to empower routine AMR surveillance data understanding and sharing.”

The sentence below is added in the “Statistical Analysis” section under the “Methods” to clarify the variables included in the multivariable analysis:

“Multivariable mixed-effect models were also performed to control for other variables, including blood culture utilization rates of the hospitals and the health region where the hospitals located.”

Reviewer #3: No further comments at this stage. Authors have tried to address largely the concerns raised earlier.

Reviewer #4: All the previous comments have been addressed but the author have not mentioned which AMR proportions and Mortalities were analyzed using either the chi square test or fisher exact test or both.

Response: We thank the reviewer for highlighting the confusing point on Chi square test and Fisher exact test. In the final analysis, neither Chi-square tests nor Fisher Exact tests were used. We have revised the sentence below to avoid confusion:

“We preliminarily compared AMR proportions and mortality using Chi-square or Fisher’s exact test when small samples (i.e. one or more expected values was <5 observations) and measurements from continuous variables using the Krustal-Wallis test. Then, we ..”

We also add all statistical tests used for all footnotes for clarity as below:

Footnote of Figure 1: “Comparison was made using the Kruskal-Wallis test.”

Footnote of Figure 2: “Comparison was made using mixed-effect logistic or Poisson regression models of patients nested within hospitals.”

Footnote of Figure 3: “Comparison was made using mixed-effect logistic or Poisson regression models of patients nested within hospitals.”

All footnotes of supplementary tables.

line 222: author repeatedly mentioned "estimate of magnitude" but there is no mention of coefficient in result section , figures or in the supplemental material. kindly provide the output of analysis in stata showing coefficient.

Response: The coefficients (from both univariable and multivariable models) are reported in the main text and supplementary materials in the form of incidence rate ratio (IRR) and mortality rate ratio (MRR), which are the exponentials of the coefficients. We decided not to report odds ratio of the logistic regression models to avoid confusion. We also removed the word “magnitude” to avoid confusion.

7. PLOS authors have the option to publish the peer review history of their article ([what does this mean?](https://url.uk.m.mimecastprotect.com/s/wrAvCNEDPFBLZMsrlY97?domain=journals.plos.org)). If published, this will include your full peer review and any attached files.


If you choose “no”, your identity will remain anonymous but your review may still be made public.


**Do you want your identity to be public for this peer review?** For information about this choice, including consent withdrawal, please see our [Privacy Policy](https://url.uk.m.mimecastprotect.com/s/BzmBCOElQFKYN2U5tCug?domain=plos.org).

Reviewer #1: No

Reviewer #3: No

Reviewer #4: No

***All contributing authors have reviewed and concurred with the revised manuscript.***

***Yours,***

***Direk Limmathurotsakul***
