# Supplementary material for: Frequency and mortality rate following antimicrobial-resistant bloodstream infections in tertiary-care hospitals compared with secondary-care hospitals
Source: PLoS One. 2024 May 20;19(5):e0303132. doi: 10.1371/journal.pone.0303132 (PMC11104583; doi:10.1371/journal.pone.0303132)
Supplement: Attachment — Submitted filename: response_to_reviewers_3_DL.docx [file pone.0303132.s006.docx]

***Re: PONE-D-23-31373R2***

***Frequency and mortality rate following antimicrobial-resistant bloodstream infections in tertiary-care hospitals compared with secondary-care hospitals***

***PLOS ONE***

***Dear Editor and Reviewers,***

***We would like to thank the editor and all the reviewers for their time reading and commenting on our manuscript. We have revised the manuscript based on the feedback.***

***We very much appreciate the suggestions and made changes in our manuscript as below:***

Reviewer #1: I appreciate the research team for answering the concerns. The authors' response does address some of the concerns by providing clarification on the nature of the data and the purpose of the study. However, there are still aspects that could be further explicit to improve the clarity and robustness of the study.

Demographic Variables: The authors clarify that individual patient-level data on demographic variables was not collected and that the study relied on aggregated statistics. While this explanation addresses the absence of demographic variables, it would be beneficial for the authors to explain why such data was not collected or why it was not deemed necessary for the model.

***Response: We are grateful for the advice. We have revised and elaborated the details of data collected from the study hospitals and used for this study in the method section as follow, “Variables in the microbiology data file included patient hospital number (HN), specimen type, specimen collection date, culture result, and antibiotic susceptibility testing result, and each row contained information for each specimen. Variables in the hospital admission data file included HN, admission date, discharge date, and in-hospital discharge outcome, and each row contained information for each admission.”***

***We have also revised and elaborated this limitation in the discussion section as follow, “These data were not available because our study was not designed to collected those data. The aim of this study was to examine the burden of AMR BSI observed in TCHs and compare that observed in SCHs using AMASS.^15^ To understand the size of effect caused by the difference in hospital type alone, further studies using detailed clinical data, AMS and IPC data, and rigorous statistical approaches are needed.”***

Confounding Variables: The authors mention that multivariable mixed-effect models were performed to control for other variables, including blood culture utilization rates of the hospitals and the health region where the hospitals were located. While this adds some clarity regarding the confounding variables, it would be helpful for the authors to elaborate on why these specific variables were chosen and how they were determined to be relevant to the analysis. Additionally, providing information on any other potential confounders that were considered but not included in the analysis would strengthen the robustness of the study.

***Response: We are grateful for the advice. We have revised and elaborated the details of data collected from the study hospitals in details in the method section as follow, “We adjusted for blood culture utilization rate because if blood culture utilization rate was low, the observed AMR proportions could be higher than the true susceptibility profiles of pathogenic organisms.^22^ Additionally, the observed AMR frequency per 100,000 admissions and per 100,000 patient-days could be lower than the true incidence rate of AMR infections.^22^ We adjusted for health regions to control for potential variations in the AMR proportions and frequency across different regions. These regional differences could be influenced by other factors such as habits of antibiotic use and economic levels which were not explored in this study. There were no other potential confounders that were evaluated in the analysis.”***

***All contributing authors have reviewed and concurred with the revised manuscript.***

***Yours,***

***Direk Limmathurotsakul***
